# Osteopontin Expression in Small Airway Epithelium in Copd is Dependent on Differentiation and Confined to Subsets of Cells

**DOI:** 10.1038/s41598-019-52208-3

**Published:** 2019-10-29

**Authors:** Mohamad N. Ali, Michiko Mori, Tinne C. J. Mertens, Premkumar Siddhuraj, Jonas S. Erjefält, Patrik Önnerfjord, Pieter S. Hiemstra, Arne Egesten

**Affiliations:** 1 Respiratory Medicine & Allergology, Department of Clinical Sciences Lund, Lund University and Skåne University Hospital, Lund, Sweden; 2 Rheumatology & Molecular Skeletal Biology, Department of Clinical Sciences Lund, Lund University and Skåne University Hospital, Lund, Sweden; 3 Unit of Airway Inflammation, Department of Experimental Medical Sciences, Lund University and Skåne University Hospital, Lund, Sweden; 4 Department of Pulmonology, Leiden University Medical Center, Leiden, The Netherlands; 5 Present Address: Department of Biochemistry and Molecular Biology, University of Texas Health Science Center at Houston, Houston, TX USA

**Keywords:** Translational research, Respiratory tract diseases

## Abstract

Osteopontin (OPN) plays a role in inflammation via recruitment of neutrophils and tissue remodeling. In this study, we investigated the distribution of OPN-expressing cells in the airway epithelium of normal lung tissue and that from patients with chronic obstructive pulmonary disease (COPD). OPN was detected on the epithelial cell surface of small airways and in scattered cells within the epithelial cell layer. Staining revealed higher OPN concentrations in tissue showing moderate to severe COPD compared to that in controls. In addition, OPN expression was confined to goblet and club cells, and was absent from ciliated and basal cells as detected via immunohistochemistry. However, OPN expression was up-regulated in submerged basal cells cultures exposed to cigarette smoke (CS) extract. Cell fractioning of air-liquid interface cultures revealed increased OPN production from basal compartment cells compared to that in luminal fraction cells. Furthermore, both constitutive and CS-induced expression of OPN decreased during differentiation. In contrast, cultures stimulated with interleukin (IL)-13 to promote goblet cell hyperplasia showed increased OPN production in response to CS exposure. These results indicate that the cellular composition of the airway epithelium plays an important role in OPN expression and that these levels may reflect disease endotypes in COPD.

## Introduction

Osteopontin (OPN) is a highly anionic phosphoglycoprotein whose sequence contains integrin-binding motifs^1^. It was originally isolated from bone and later identified in several organs, including the kidneys, pancreas, and lungs^2^. OPN is secreted by a variety of cells, such as activated T cells, natural killer (NK) cells, dendritic cells, and macrophages^3–5^. During inflammation, it is involved in cell-mediated immunity, neutrophil recruitment, regulation of dendritic cell activity, interaction with innate antibiotics, and promotion of fibrosis in lung injury^6–10^. OPN is increased in airway secretions during chronic airway inflammation and is detectable at high concentrations in sputum and bronchoalveolar fluid in diseases such as asthma, cystic fibrosis, and chronic obstructive pulmonary disease (COPD)^11–14^.

COPD is characterized by irreversible airflow limitation due to chronic airway inflammation. Cigarette smoking is a major risk factor for the development and progression of COPD in industrialized societies^15^. This heterogeneous disease comprises several phenotypes including the sub-type chronic bronchitis, which is characterized by increased mucus discharge and re-modeling of the airway epithelial lining (i.e., goblet cell hyperplasia)^16^. *In vitro* and *in vivo* studies comparing smoking to non-smoking asthmatics have shown that cigarette smoke (CS) increased OPN production in the airways^12,17,18^. Furthermore, OPN contributed to airway matrix remodeling, an important event in COPD progression^19–21^. Another feature of COPD is prolonged and dysregulated inflammation, in which the epithelium plays key roles in neutrophil recruitment and macrophage activation, thus leading to excessive protease activity and the development of emphysema^16,22^.

Several lines of evidence suggest the key role of OPN in the events leading to the development of COPD. However, to date, the cells responsible for OPN production in the airway epithelium have not been identified. In this study, we characterized OPN-producing cells in the small airways of normal lung tissue and at different stages of COPD progression. In addition, the impact of airway epithelium differentiation and CS exposure on OPN expression was investigated in primary airway epithelial cell cultures. Our results indicate that OPN levels may reflect disease endotypes in chronic airway inflammation.

## Materials and Methods

### Patients and lung tissue samples

Macroscopically normal, tumor-free lung tissue samples were obtained during transplantation from patients undergoing cancer surgery. The clinical phenotypes of the individuals are listed in Table S1. All patients were aged >18 years and provided written informed consent to participate in this study, which was approved by the Regional Ethical Review Board in Lund (approval no. LU412-03). All experiments were performed in accordance with the Declaration of Helsinki as well as relevant guidelines and regulations.

### Immunocytochemistry and immunohistochemistry (IHC)

Immediately after collection, lung tissue samples were placed in 4% buffered formaldehyde. After dehydration and embedding in paraffin, thin sections (3 μm) were produced.

#### Staining for p63, mucin 5AC (MUC5AC), and uteroglobin (UTG) in submerged cells

Human bronchial epithelial cells (HBECs, Lonza/Fischer Scientific, Göteborg, Sweden) were seeded on poly-L lysine-coated glass coverslips, placed in a 24-well plate, and maintained in bronchial epithelium cell medium (BEpiCM, ScienCell, Carlsbad, CA, USA) in a 5% CO_2_ incubator at 37 °C until 80–90% confluence. After washing and fixation in 4% paraformaldehyde, cells were permeabilized using Triton X-100 (0.1% in phosphate-buffered saline, PBS). This was followed by washing, blocking with 5% bovine serum albumin (BSA) in PBS with Tween® 20 (PBST), and labeling with a murine monoclonal antibody against p63 (1:250; ab735, Abcam, Cambridge, UK). This was visualized after incubation at room temperature (RT) for 1 h with an Alexa Fluor 594-conjugated goat anti-mouse secondary antibody (1:500; Thermo Fischer Scientific, Waltham, MA, USA). A primary murine monoclonal antibody against MUC5AC was used (1:250; MA1-38223, Invitrogen, Carlsbad, CA, USA) and visualized using the method described for detection of p63. Nuclei were stained using 4′,6-diamidino-2-phenylindole (DAPI; Prolong Gold antifade reagent with DAPI, Thermo Fisher Scientific).

#### Single staining of OPN

A single staining protocol (EnVision™ Detection system, K5007, Dako, Glostrup, Denmark) was used for visualization of OPN. Briefly, after antigen retrieval (cat. no. K8005, Dako), OPN was detected using rabbit anti-OPN antibodies (1:800; generously provided by the late Professor Dick Heinegård, Lund) and visualized using secondary goat anti-rabbit antibodies conjugated with peroxidase polymers (Dako). These IHC protocols were performed using an automated IHC robot (Autostainer Plus, Dako). Sections were counter-stained with Mayer’s hematoxylin for visualization of background tissue, dehydrated in alcohol/xylene, and mounted on Pertex (Histolab, Göteborg, Sweden).

#### Double staining using immunofluorescence

In the case of immunofluorescence staining, three COPD GOLD stage IV donors (2–4 samples per donor), two COPD GOLD stage II donors (2 samples per donor) and three never smoking controls (2 samples per donors) were investigated and representative micrographs were chosen for the figures. In general, there was in the order of five small airways per tissue section. After antigen retrieval (cat. no. K8005, Dako) and protein blocking (cat. no. X0909, Dako), sections were incubated for 1 h at RT with rabbit anti-OPN antibodies (1:80) and immunoreactivity was visualized after incubation for 1 h at RT with a donkey anti-rabbit Alexa Fluor 647-conjugated secondary antibody (1:200; Thermo Fischer Scientific). In subsequent staining, sections were incubated with a murine monoclonal antibody against MUC5AC (1:100; cat. no. MA1-38223, Thermo Fisher Scientific) and visualized after incubation at RT for 1 h with an Alexa Fluor 488-conjugated goat anti-mouse secondary antibody (1:200; Thermo Fischer Scientific). Consecutive sections were stained using a monoclonal rat anti-UTG antibody (1:150; cat. no. MAB4218, R&D Systems, Minneapolis, MN, USA) and visualized after incubation with an Alexa Fluor 488-conjugated goat anti-rat secondary antibody (1:200; Thermo Fischer Scientific), a monoclonal murine antibody against p63 (1:50; cat. no. ab735; Abcam) and visualized after incubation at RT for 1 h with an Alexa Fluor 488-conjugated goat anti-mouse secondary antibody (1:200; Thermo Fischer Scientific), or polyclonal goat antibodies against forkhead box protein J1 (FOXJ1, 1:250; cat. no. AF3619, R&D Systems) visualized using donkey anti-goat using Alexa Fluor 555-conjugated secondary antibodies (1:200; Thermo Fischer Scientific). Nuclei were visualized using Hoechst stain (H33342; Sigma-Aldrich, St Louis, MO, USA), and sections were mounted in Tris-buffered saline (TBS)/glycerin and frozen until quantification. Isotype-matched control antibodies (Dako) were used to replace primary antibodies in all unstained IHC procedures for markers and tissues.

### Histochemical staining

Inserts from air-liquid interface (ALI)-cultures were placed in 4% buffered formaldehyde. After dehydration and embedding in paraffin, thin sections (3 μm) were produced. These were dipped in distilled water before staining with alcian blue (Histolab) for 15 min. Sections were then washed under running tap water for 2 min followed by washing with distilled water. The sections were counter-stained with Mayer’s hematoxylin (Sigma-Aldrich), dehydrated in alcohol/xylene, and mounted on Pertex (Histolab).

### Staining intensity analysis

High-resolution digital images of whole slides were generated using a ScanScope Slide Scanner (Aperio Technologies, Vista, CA, USA). In each small airway (<2 mm cross-sectional internal diameter, non-cartilaginous), the epithelium was manually delineated and the total number of pixels of OPN-immunoreactive cells (showing brown deposits as a result of 3,3′-diaminobenzidine (DAB) reacting with immobilized peroxidase-conjugated antibodies) was calculated and normalized to the number of pixels of the entire airway epithelium area using Aperio Positive Pixel Count Algorithm v.9 (Aperio Technologies).

### CS extract (CSE)

Aqueous CSE was prepared using the method described by Blue and Janoff^23^, with modifications. A smoking apparatus (50-mL plastic syringe with a three-way stopcock) was used, to which a cigarette (Kentucky 1RF4 cigarettes, Kentucky Tobacco Research & Development Center, Lexington, KY, USA) and a sterile plastic tip were attached. Via the plastic tip, 35 mL CS was slowly bubbled into 30 mL sterile RPMI 1640 for 30 min. One cigarette was used per 10 mL medium. The CSE solution was then filtered, aliquoted, and stored at −80 °C until further use.

### Airway epithelial cell culture

#### Submerged cell cultures

Human bronchial epithelial cells (HBEpiCs/HBECs, ScienCell and Lonza/Fischer Scientific) were cultured in BEpiCM (ScienCell) at 37 °C in an atmosphere containing 5% CO_2_ until 80–90% confluence. Next, cells were washed once with PBS, and treated with trypsin (ACF Enzymatic Dissociation Solution, Stemcell Technologies, Vancouver, Canada) followed by incubation at 37 °C in 5% CO_2_ for 5 min. Once the cells were dissociated, an equal volume of trypsin solution was added, followed by centrifugation at 230 × *g* for 5 min before aspirating the medium and re-suspending the cell pellet in 1 mL fresh medium. For experiments, 24-well plates were coated with poly-L-Lysine (ScienCell) for at least 1 h at 37 °C. Wells were washed once with Dulbecco’s PBS before seeding the cells (30 000 cells/well). Upon reaching 80% confluence, cells were stimulated with 5% (v/v) CSE for 24 h at 37 °C. At the end of the incubation period, plates were centrifuged at 230 × *g* for 5 min before collecting cells and supernatants for further experiments.

#### ALI cultures

Primary bronchial epithelial cells (PBECs) were obtained from tumor-free resected lung tissue at Leiden University Medical Center, Leiden, the Netherlands, and cultured to achieve mucociliary differentiation at an ALI, as described previously^24,25^. Briefly, PBECs at passage 2 were cultured submerged on semipermeable, 0.4-μm Transwell inserts (Corning Costar, Cambridge, MA, USA) coated with a mixture of BSA, collagen type 1, and fibronectin. Once fully confluent, the apical medium was removed and PBECs were cultured for at least 2 weeks to achieve mucociliary differentiation before use in subsequent experimental procedures.

### Chronic whole CS exposure

To assess the effect of chronic CS exposure on differentiating PBECs, cells were exposed daily to either air alone or to whole CS from the time of culture start at the ALI till day 14, followed by a further 5 days of air-only exposure. Briefly, apical medium was aspirated and ALI-PBECs were washed with PBS. After 4 h, cells were exposed to whole CS for 4–5 min in a hypoxic chamber before flushing the chamber with air for 10 min and returning the cells to the incubator^25^. The same procedure was repeated after approximately 18–20 has previously described^26^, and this cycle was continued for 14 days^25^.

### Enzyme-linked immunosorbent assay (ELISA)

OPN levels in the cell culture media of bronchial epithelial cells were determined via ELISA (Osteopontin DuoSet ELISA kit, R&D Systems) according to the manufacturer’s instructions.

### Isolation of basal and luminal epithelial cell fractions

Apical and luminal cell fractions were isolated from ALI-PBEC cultures after 19 days as previously described^26^. Briefly, cells were washed with calcium-free PBS before adding calcium-free minimum essential medium (MEM, Thermo Fischer Scientific) to both the apical and basolateral compartments to compromise the integrity of the intercellular junctions. Next, the apical medium was substituted for Trypsin Versene (Thermo Fischer Scientific) and incubated for 7–10 min. Detached cells were collected as the luminal fraction while cells still attached to the inserts were harvested as the basal fraction.

### *In situ* hybridization for detection of OPN gene expression in ALI cultures

Inserts from ALI-PBECs were collected and fixed with 4% paraformaldehyde followed by dehydration and embedding in paraffin. Thereafter, 3–4 μm sections were produced and mounted on glass slides. The sections were treated with RNAscope Protease Plus reagent (Bio-Techne, Minneapolis, MN, USA) before boiling in RNAscope Target Retrieval (Bio-Techne) for 15 min. After washing, a mixture of Oligo Probes against OPN mRNA (Bio-Techne) was added to the sections followed by incubation for 2 h in a HybEZ^TM^ II Oven (Bio-Techne). Subsequently, a series of amplification probes (RNAscope 2.5 HD Detection Reagent-RED, Bio-Techne) were added to the sections for hybridization with a cascade of signal amplification molecules. These were then detected using a Fast RED-A and Fast RED-B reagent mixture (Bio-Techne). Finally, the slides were counterstained, washed, and visualized via light microscopy.

### RNA isolation, cDNA synthesis, and quantitative polymerase chain reaction (qPCR)

Cells were washed once with PBS before lysis and collection. RNA was isolated using an illustra^TM^ RNAspin Mini kit (GE Healthcare Life Sciences, Chicago, IL, USA) according to the manufacturer’s instructions. RNA was quantified using a Nanodrops ND-1000 Spectrophotometer (Saveen and Werner, Malmö, Sweden). cDNA was analyzed using an iScript^TM^ cDNA Synthesis Kit (Bio-Rad, Hercules, CA, USA). Real-time qPCR analysis was performed using the primers listed in Table S2 and iTaq^TM^ Universal SYBR Green Supermix (Bio-Rad). Reactions were performed in triplicate and the results were corrected using the mean expression of reference genes. Values were normalized and plotted as fold-changes.

### Effects of interleukin (IL)-13 and CS on OPN expression

The effects of CS on OPN production in ALI-PBECs differentiated in the presence of IL-13, which promotes goblet cell hyperplasia, were investigated in this experimental model. PBECs were grown to confluence and cultured at the ALI for 14 days in the presence and absence of recombinant human IL-13 (2.5 ng/mL, Peprotech, Rocky Hill, NJ, USA), which was added to the basolateral compartment of Transwell inserts. This was followed by exposure to whole CS or air once daily for a further 5 days in the presence and absence of continued IL-13 treatment (2.5 ng/mL), which was added to the basolateral compartment of the wells. Medium was refreshed every 2 days during the first 14 days and daily during the last 5 days, directly after whole CS or air exposure. Medium from the basolateral compartment and cells from the apical compartment were collected 24 h after final exposure to whole CS or air^25^.

### Statistical analyses

Data were expressed as means and standard deviations (SD) and were analyzed using Student’s *t***‐**test (parametric and nonparametric). For statistical evaluation of more than two experimental groups, one-way analysis of variance (ANOVA) was used (followed by Tukey’s and Dunnett’s post-hoc tests). All statistical evaluations were performed using GraphPad Prism software 8.0 (GraphPad Software, La Jolla, CA, USA). Differences were considered significant if *P* < 0.05.

## Results

### Distribution of OPN in lung tissue from patients with COPD and controls

**Figure 1:**
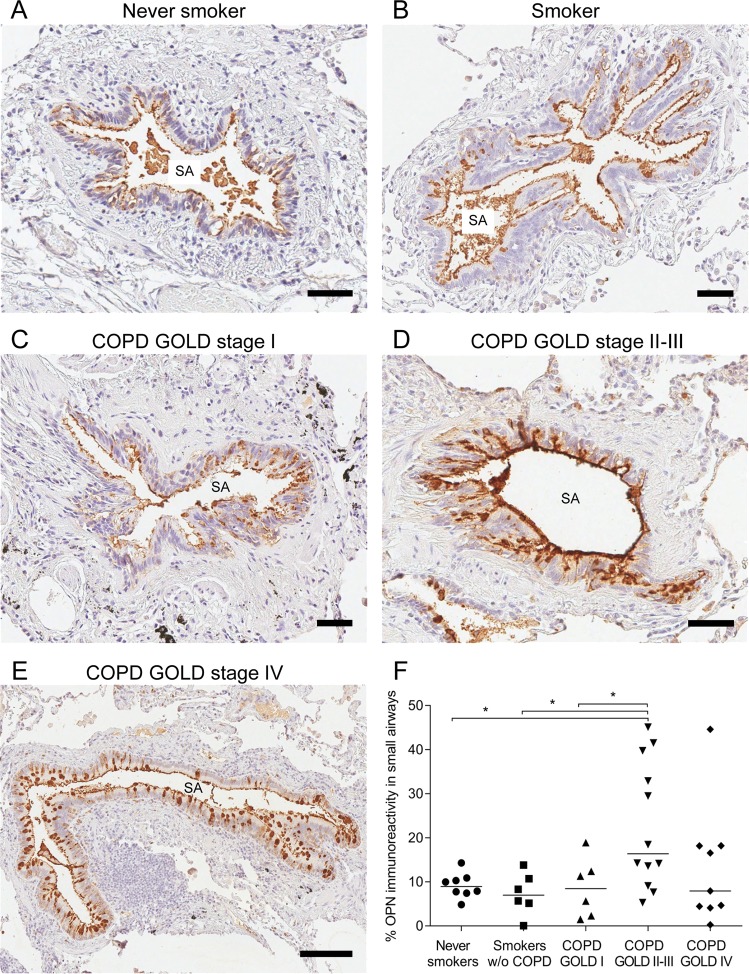
Localization of osteopontin (OPN) in small airways. Immunoreactivity for OPN was visualized via reaction with 3,3′-diaminobenzidine (DAB), resulting in brownish staining. OPN was observed on the apical surface of the airway mucosa as well as in cells scattered within the epithelial cell layer of small airways in a never-smoker **(A)**, smoker without chronic obstructive pulmonary disease (COPD) **(B)**, and patients with COPD at various stages of the Global Initiative for Chronic Obstructive Lung Disease (GOLD) classification **(C–E)**. Analysis of immunoreactivity within the epithelial cell layer was performed and groups were compared via one-way analysis of variance (ANOVA) with Dunnett’s post-hoc test. **(F)** *P < 0.05. SA = Small airway. Scale bars: A–D = 50 µm, E = 150 µm.

IHC staining was performed on sections of lung tissue obtained from smokers (with or without COPD) and non-smokers (Supplemental Table I) to determine OPN distribution in small airways. COPD severity was staged according to the Global Initiative for Chronic Obstructive Lung Disease (GOLD) classification using the forced expiratory volume in 1 s (FEV_1_) after bronchodilation as previously described^27^. Staining analysis revealed that OPN was constitutively expressed in the epithelium of small airways (Fig. 1), showing positive staining on the apical lining of the airway mucosa as well as in the cytoplasm of scattered cells within the airway epithelium. Staining intensity was compared in never-smokers, current smokers, and patients with different stages of COPD, and was significantly higher in patients at GOLD stage II/III compared to those in other groups, but not in GOLD stage IV (Fig. 1F).

### OPN was expressed in goblet and club cells of the airway epithelium

**Figure 2:**
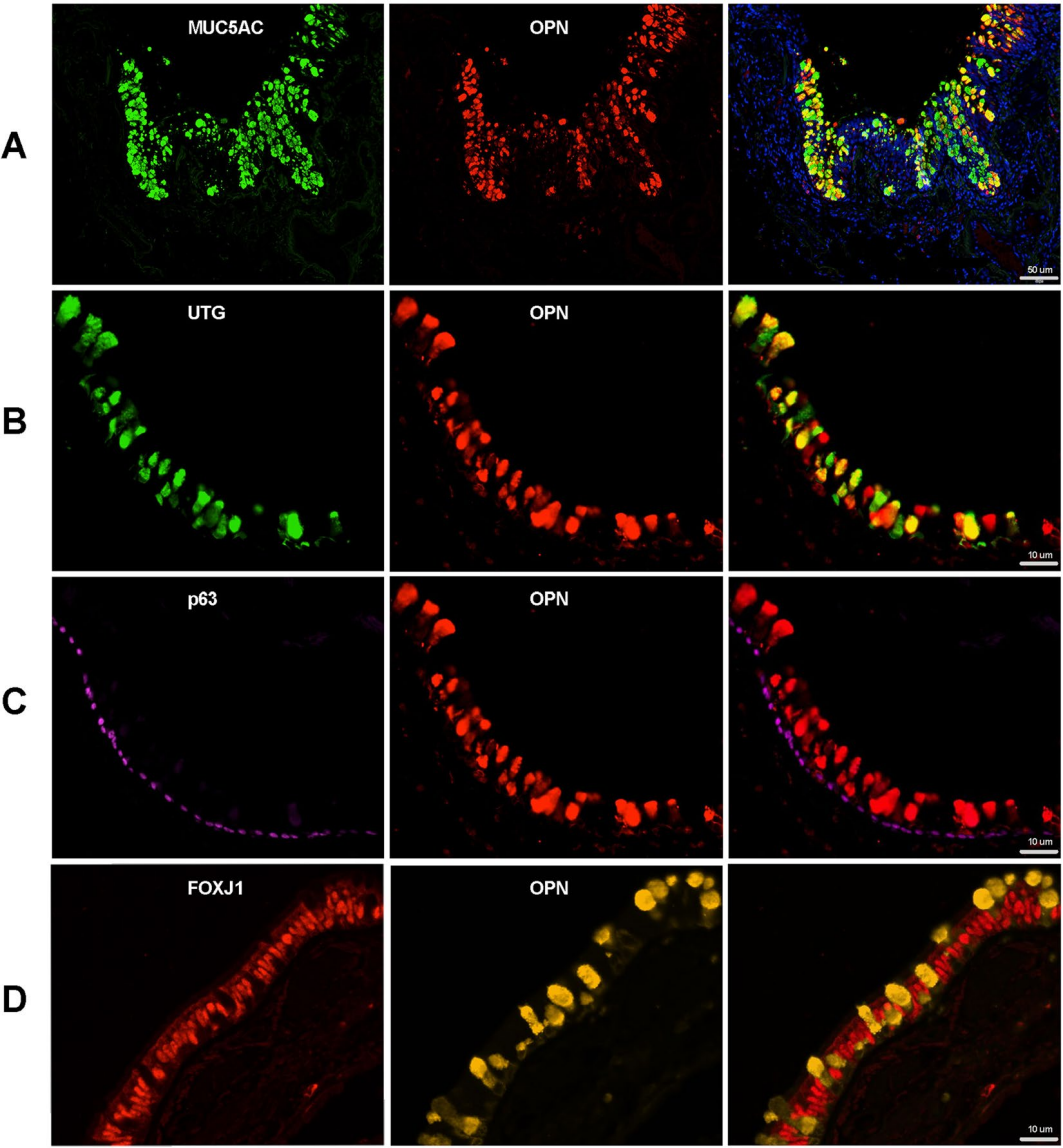
Phenotypic characterization of OPN-expressing cells in small airways during COPD (GOLD stage II). Immunofluorescence was used to detect possible co-localization of OPN and mucin 5AC (MUC5AC) in goblet cells **(A)**, uteroglobin (UTG) in club cells **(B)**, p63 in basal cells **(C)**, and forkhead box protein J1 (FOXJ1) in ciliated cells **(D)**. In the overlay, a high degree of co-localization was observed for OPN and MUC5AC in goblet cells and UTG in club cells. Scale bars: A = 50 µm, B–D = 10 µm.

To characterize the phenotype of OPN-expressing cells, all cells were double-stained using immunofluorescence (Fig. 2). OPN co-localized to a high degree with MUC5AC in goblet cells (Fig. 2A), and with UTG in club cells (Fig. 2B), but not with p63 in basal cells (Fig. 2C) or FOXJ1 in ciliated cells (Fig. 2D). Similar results were obtained in tissue of COPD GOLD stage II and IV as well as in never smoking controls (Supplemental Fig. 1).

### CSE increased OPN expression in submerged cultures of bronchial epithelial cells

**Figure 3:**
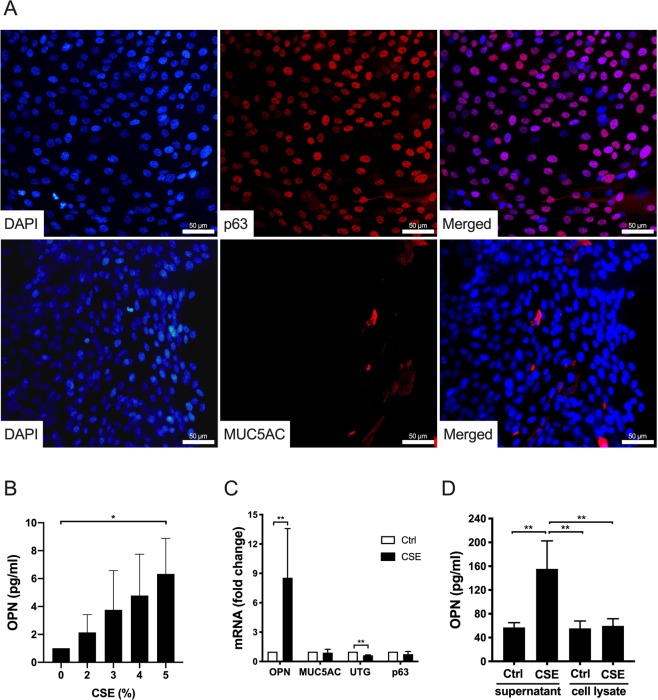
Phenotypic characterization of submerged human bronchial epithelial cells (HBECs) and OPN production in response to cigarette smoke extract (CSE). **(A)** HBECs were grown to near confluence and stained to detect markers of basal cells (p63) and goblet cells (MUC5AC). 4′,6-Diamidino-2-phenylindole (DAPI) was used to stain the DNA of nuclei. Micrographs from one representative experiment out of three. Scale bars = 50 µm. **(B)** HBECs were incubated with CSE (0–5%) for 24 h. OPN levels in cell culture media were determined via enzyme-linked immunosorbent assay (ELISA). Results represent means and standard deviations (SD) of three independent experiments. Statistical analyses were performed using one-way ANOVA with Dunnett’s post-hoc test. **(C)** Submerged HBECs were cultured in the absence and presence of CSE (5%) for 24 h. The mRNA expression of OPN, MUC5AC, UTG, and p63 (fold-change compared to control cells cultured in medium alone) is depicted here. The results represent means and SD of three to five independent experiments. Statistical analyses were performed using a Mann-Whitney U test. **(D)** Intracellular OPN content and content in the media of cells cultured in the absence and presence of CSE (5%) for 24 h. The results represent means and SD of three independent experiments. Statistical analyses were performed using one-way ANOVA with Tukey’s post-hoc test. *P < 0.05, **P < 0.01.

CS is a potent inducer of OPN expression in cultured alveolar macrophages, *in vivo* models of emphysema, and smoking asthmatics patients^12,17,18^. To investigate OPN expression in airway epithelial cells, submerged cultures of normal HBECs were exposed to CSE. Submerged HBECs are likely to retain an undifferentiated phenotype with a majority of p63-positive cells, which is characteristic of basal cells (Fig. 3A). In contrast, neither MUC5AC (Fig. 3A) nor UTG (not shown) was detected. Incubation of submerged HBECs with increasing concentrations of CSE resulted in a dose-dependent up-regulation of OPN production, reaching a maximum at a concentration of 5% (Fig. 3B). Cytotoxicity, measured as lactate dehydrogenase (LDH) release, was detected at CSE concentrations ≥ 7% (data not shown). This increase in OPN gene expression in response to CSE exposure (5%) was further confirmed via qPCR. As IHC on lung sections revealed the presence of OPN in goblet and club cells (Fig. 2), qPCR was performed to verify that CSE did not induce differentiation of basal cells towards these cellular phenotypes, thus explaining the increased production of OPN. RNA was isolated from submerged cells before and after 24 h stimulation with CSE (Fig. 3C). However, no significant changes were observed with regard to MUC5AC and p63, although UTG expression significantly decreased after CSE stimulation. This indicates that OPN expression in basal cells increased upon stimulation with CSE, without causing differentiation into goblet or club cells. Immunocytochemistry could not be performed on cells after CSE stimulation because of significant background staining. Because IHC did not reveal significant staining for OPN in basal cells of the airway epithelium *in vivo*, HBECs in medium alone and those stimulated with CSE (5%) were lysed using radioimmunoprecipitation assay (RIPA) buffer (Thermo Fisher Scientific) and intracellular OPN content was measured in parallel to that in the medium via ELISA (Fig. 3D). A statistically significant increase in OPN concentrations was observed in the medium after CSE stimulation although low OPN levels in cell lysates remained stable when comparing resting and CSE-stimulated cells. This suggests that basal cells do not store but rather continuously release OPN produced, which may explain low OPN staining in basal cells *in vivo*.

### OPN expression and production in luminal and basal cell fractions of ALI cultures

**Figure 4:**
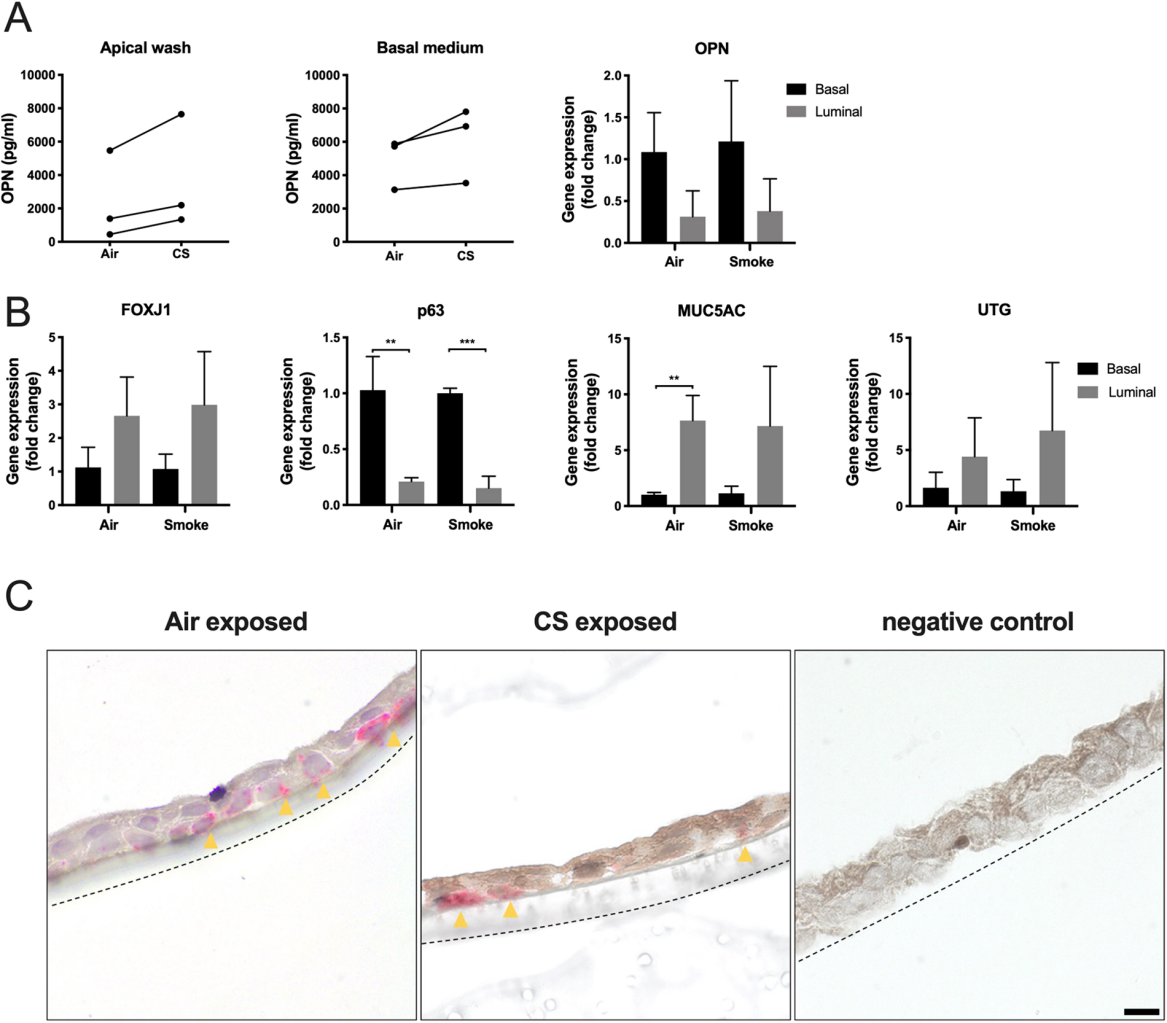
Luminal and basolateral OPN expression and release in air-liquid interface (ALI) cultures. After 14 days of differentiation, ALI cultures were exposed to either air or CS (from one cigarette daily) for 5 consecutive days. The cells were separated into basal and luminal fractions. **(A)** OPN content in apical washes and basal medium was determined via ELISA. Quantitative polymerase chain reaction (qPCR) was performed to compare OPN expression in basal and luminal fractions, in air- and CS-exposed cultures. Fold-changes were calculated to compare gene expression in basal and luminal cells using parametric Student’s *t***‐**tests. **(B)** The following markers for subsets of airway epithelial cells in basal and luminal fractions were investigated via qPCR: FOXJ1 (ciliated cells), p63 (basal cells), MUC5AC (goblet cells), and UTG (club cells). Data were analyzed using parametric Student’s *t***‐**tests. *P < 0.05 and **P < 0.01. **(C)** *In situ* hybridization was performed on sections obtained from ALI cultures exposed to air or CS. Yellow arrows indicate the site of hybridization to OPN mRNA (red dots). The dotted lines represent insert membranes. The negative control did not contain RNA-specific oligo probes. Scale bar = 10 µm.

As OPN production is confined to subsets of airway epithelial cells, the distribution of cellular phenotypes may determine how OPN is released. To investigate this, cells from differentiated ALI cultures were exposed to either air or CS, separated into basal and luminal cell fractions, and subjected to qPCR to compare OPN expression (Fig. 4A). A non-significant increase in expression was observed in basal cell fractions compared to that in luminal cell fractions after both air and CS exposure. OPN concentrations in washes from the apical cell surfaces were compared to OPN content in basal medium via ELISA (Fig. 4A), and the basolateral release of OPN was comparable or slightly higher. A tendency towards higher OPN expression in the basal cell fraction, as detected by qPCR, could suggest that there is a polarized secretion of OPN to the apical surface from cells in this compartment. OPN levels were increased after CS exposure in all donors, although large donor-dependent variations were observed (Fig. 4A). To confirm the presence of phenotypic subsets in each fraction, specific markers were investigated via qPCR, comparing air and CS exposure (Fig. 4B). p63 expression was significantly higher in basal cell fractions than in luminal fractions after both air and CS exposure, thus confirming basal cell enrichment. In contrast, MUC5AC expression was significantly higher in luminal fractions during air but not CS exposure. Expression of FOXJ1 and UTG was slightly higher in luminal fractions than that in basal fractions, although this was not statistically significant. *In situ* hybridization was used to detect OPN-expressing cells in ALI cultures exposed to air or CS (Fig. 4C). Scattered OPN-expressing cells were observed mainly in the basal region and associated with insert membranes. This may indicate the association of OPN expression to basal phenotype cells, at least to some extent. For technical reasons, it was not possible to perform double-labeling experiments to further characterize the phenotypes of these cells. Similarly, *in situ* hybridization could not be carried out on lung tissue because of poor mRNA stability.

### ALI culture differentiation and OPN production in response to CS and IL-13 stimulation

**Figure 5:**
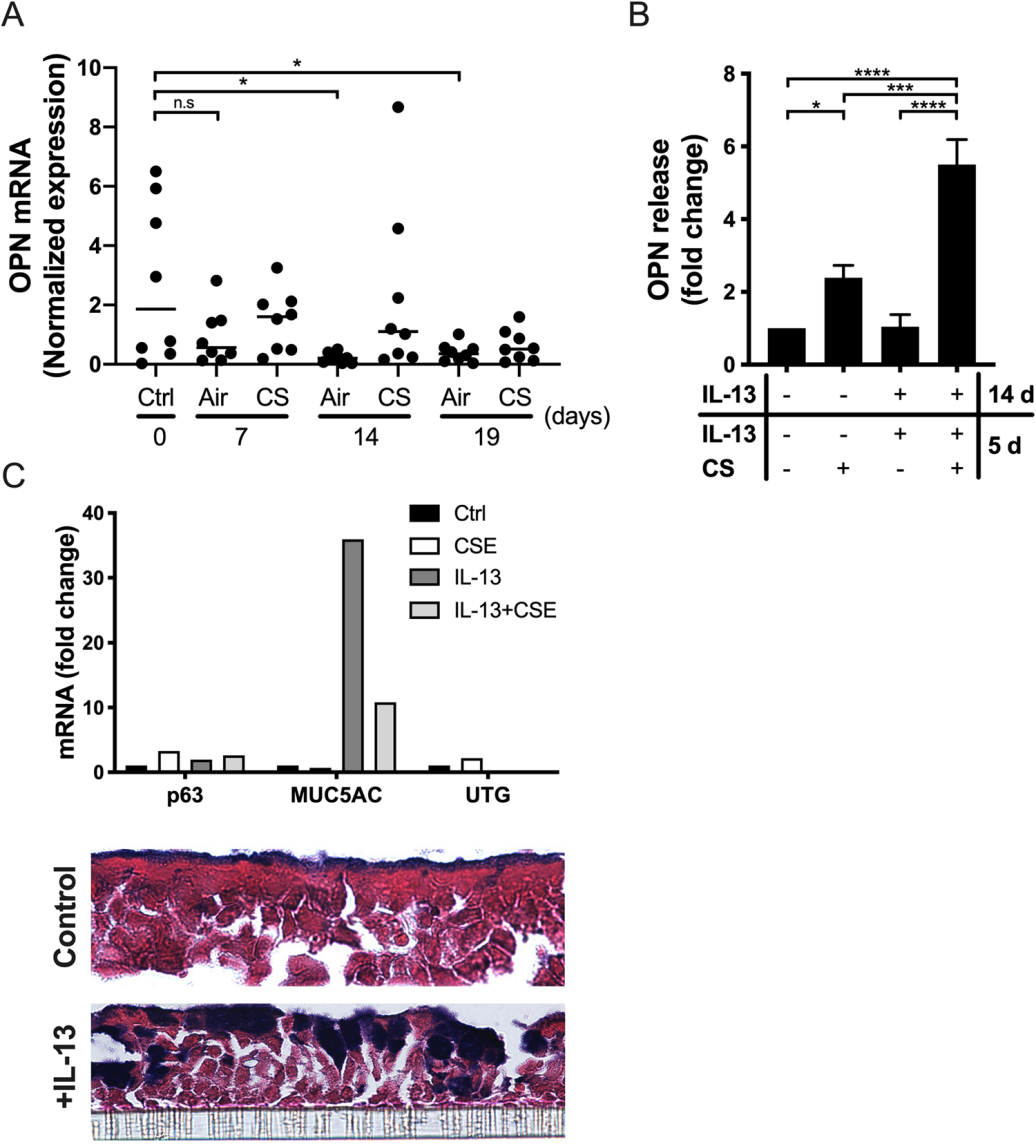
OPN expression in ALI cultures during differentiation and effect of interleukin (IL)-13. **(A)** Primary bronchial epithelial cells (PBECs) were exposed daily to whole CS, one cigarette per day, for 14 consecutive days followed by an additional 5 days of air-only exposure. Cells were collected on days 0, 7, 14, and 19 for RNA isolation and qPCR to assess OPN gene expression. Data shown were normalized to the expression of a house-keeping gene (ribosomal protein (RP) L13A; n = 8 donors). Bars represent mean values in each group. Statistical analyses were performed using one-way ANOVA with Dunnett’s post-hoc test. **(B)** Cells were grown to confluence before air exposure and cultured for 14 days in the absence and presence of IL-13 (2.5 ng/mL), followed by once-daily whole CS or air exposure for an additional 5 days in the continued absence or presence of IL‐13. Media were collected and OPN levels were determined via ELISA. Data shown were normalized to OPN production in cells grown in medium alone (set to 1; n = 3 donors). Statistical analyses were performed using one-way ANOVA with Tukey’s post-hoc test. **(C)** HBECs were grown to confluence before air exposure and cultured for 14 days in the absence and presence of IL-13 (2.5 ng/mL), followed by the addition of CSE (5%). After 24 h, RNA was isolated to assess p63, MUC5AC, and UTG gene expression and inserts were paraffin-embedded, sectioned, and stained with alcian blue to detect mucins in goblet cells (purple-blue). The striped line represents the insert membrane. cross section; *P < 0.05, **P < 0.01, ***P < 0.001, ****P < 0.0001; n.s., not significant.

To investigate the effect of airway epithelial cell differentiation, and CS and IL-13 exposure on epithelial OPN production, we used samples collected during a previous study^25^. First, the effects of epithelial cell differentiation and chronic CS exposure on OPN expression were examined (Fig. 5). ALI cultures of PBECs were exposed to either air or CS from one cigarette daily for 14 days, followed by air-only exposure for an additional 5 days (Fig. 5A). CS exposure was interrupted to investigate the reversibility of OPN expression. Analysis of gene expression revealed that OPN mRNA levels decreased over time in air-exposed cells, and that this change was significant when comparing day 0 with days 14 and 19. These results may be explained by the increase in cell differentiation towards ciliated and secretory cells (including goblet cells). This is in accordance with *in vivo* results obtained via IHC, in which ciliated cells showed no OPN expression (Fig. 2). CS-exposed cells showed a slight but non-significant increase in OPN expression during differentiation. However, these levels were not significantly altered over time during differentiation (Fig. 5A). Interestingly, upon smoke cessation for 5 days, there was a non-significant decrease in OPN expression when compared to that at previous time points.

IL-13 stimulation of airway epithelial cells may trigger goblet cell hyperplasia^28,29^. To study the effects of hyperplasia on OPN production, ALI cultures were incubated in medium alone or stimulated with IL-13 for 2 weeks followed by exposure to air or CS for 5 days in the presence and absence of IL-13 (Fig. 5B). IL-13-differentiated cells were more responsive to the stimulatory effects of CS exposure over the subsequent 5 days (Fig. 5B). To confirm goblet cell hyperplasia after IL-13 stimulation, qPCR and staining with alcian blue (on inserts) were performed (Fig. 5C). High expression of MUC5AC and positive staining with alcian blue confirmed increased presence of goblet cells after stimulation with IL-13. Taken together, inducing goblet cell hyperplasia using IL-13 increased OPN secretion in response to CS exposure, an observation that is in line with previous *in vivo* results showing that MUC5AC was highly co-localized with OPN (Fig. 2). In addition, IL-13 is a key cytokine in allergic asthma and smoking allergic asthmatics show increased OPN sputum levels^9^.

## Discussion

In this study, we investigated OPN distribution in epithelial cells of the small airways and showed that staining intensity was the highest in tissue from patients with COPD, particularly those classified as GOLD stage II-III. Although it is the most severe COPD condition, no significant differences in staining intensity were observed in samples from patients with GOLD stage IV. This may be explained by advanced structural damage and remodeling of the lungs at this stage, resulting in less active disease that may in turn reduce OPN production. Goblet and club cells showed high immunoreactivity for OPN, suggesting that airway remodeling and goblet cell hyperplasia in COPD may in part explain the increased overall OPN production in this disease^30^. Furthermore, the results suggest that basal cells express and release OPN, but do not retain it.

In smokers, there is an increase in the number of goblet cells, but not club cells, in the small airways^31^. Sputum OPN levels reflect severity in asthma and higher levels are observed in smoking compared to non-smoking asthmatics^11,12^. In one study, OPN levels were significantly higher in patients with COPD than those in asymptomatic smokers and non-smokers. There was also a significant association between OPN and sputum neutrophils, IL-8, matrix metalloproteinase-2 (MMP-2), and emphysema severity^14^. Therefore, OPN levels may reflect remodeling of the airway epithelial cell layer and the resulting disease endotype as an effect of chronic airway inflammation rather than acute exacerbations.

Expression of type 2 cytokines is a typical feature of allergic asthma. However, type 2 responses are now thought to be important in COPD as well, and in particular during exacerbations^32^. In addition, IL-13 is expressed in the central airways of smokers and may contribute to mucus hypersecretion in chronic bronchitis^33^. IL-13 may therefore contribute to goblet cell hyperplasia in both asthma and COPD.

Goblet cell hyperplasia may become persistent in ex-smokers. A model comprising three stages has been proposed to explain irreversible goblet cell hyperplasia in COPD^34^. During the first phase, CS causes epidermal growth factor receptor (EGFR)-dependent reversible reprogramming of basal cells, making these cells susceptible to rhinovirus. During COPD exacerbations caused by rhinovirus infection, persistent goblet cell hyperplasia is induced via the neurogenic locus notch homolog protein 3 (Notch 3)-hairy/enhancer-of-split related with YRPW motif protein 1 (Hey1)-FOXA3 axis in basal cell-derived progenitors. Eventually, unknown mechanisms cause permanent reprogramming of basal cells resulting in goblet cell hyperplasia independent of smoking or infection. In COPD airways, these events may occur simultaneously, leading to chronic mucus hypersecretion and persistent goblet cell hyperplasia^35–37^. In addition, IL-17 associated with neutrophilic airway inflammation during COPD exacerbations may promote goblet cell hyperplasia via Notch 2 signaling in basal cells^35^. In addition to inflammation, CS may independently promote goblet cell hyperplasia by activating EGFR-signaling in basal cells^37^.

OPN contains integrin-binding motifs in its sequence and may promote neutrophil recruitment to the airways^1,38^. Furthermore, it may contribute to neutrophil-rich inflammation through binding to and neutralizing ELR-negative but not ELR-positive CXC-chemokines, thus promoting an influx of neutrophils^13^. This may be one of the mechanisms through which OPN contributes to prolonged and dysregulated inflammation during CS exposure and COPD. High basolateral OPN release, of which a large portion is derived from basal cells, may be important in the recruitment of neutrophils. Furthermore, basolateral release of OPN may be involved in tissue remodeling^20^.

Exacerbations play an important role in COPD and result in the deterioration of lung function^39^. In many cases, bacteria trigger these inflammatory bouts and innate antibiotics (i.e., antimicrobial proteins, AMPs) are important in counteracting these events^40^. However, OPN binds to and neutralizes the antibacterial activities of several AMPs expressed in the airways, including secretory leukocyte protease inhibitor (SLPI), midkine, and human β-defensin 3^8^. Consequently, inappropriate production of apically released OPN in severe COPD may contribute to exacerbations triggered by bacteria. Furthermore, OPN is thought to impair host defenses against pneumococcal airway infection in mice and humans, and CS increases the risk of severe pneumococcal disease, further associating OPN to airway infection^38,41^. In contrast, OPN protected against pneumococcal infection in a model of allergic asthma^42^. Its beneficial role was also demonstrated in a model of histone-induced acute lung injury^43^. Thus, polarized release of OPN is likely to play different roles in different contexts.

In this study, we showed that cell-associated OPN expression in small airways is confined to goblet and club cells. In addition, basal cells are likely to produce and secrete OPN, although they do not retain it. Disease-induced alteration of epithelial cell subsets, such as goblet cell hyperplasia, may affect the number of cells capable of producing OPN. However, signaling pathways that modulate OPN production in the airways and possible targets in chronic inflammation such as COPD remain to be elucidated.

## Supplementary information

Supplementary information

## Data Availability

The datasets generated and analyzed during the current study are available from the corresponding author on reasonable request.
