# Author Correction: Osteopontin Expression in Small Airway Epithelium in Copd is Dependent on Differentiation and Confined to Subsets of Cells

**DOI:** 10.1038/s41598-020-57447-3

**Published:** 2020-01-15

**Authors:** Mohamad N. Ali, Michiko Mori, Tinne C. J. Mertens, Premkumar Siddhuraj, Jonas S. Erjefält, Patrik Önnerfjord, Pieter S. Hiemstra, Arne Egesten

**Affiliations:** 1 Respiratory Medicine & Allergology, Department of Clinical Sciences Lund, Lund University and Skåne University Hospital, Lund, Sweden; 2 Rheumatology & Molecular Skeletal Biology, Department of Clinical Sciences Lund, Lund University and Skåne University Hospital, Lund, Sweden; 3 Unit of Airway Inflammation, Department of Experimental Medical Sciences, Lund University and Skåne University Hospital, Lund, Sweden; 4 Department of Pulmonology, Leiden University Medical Center, Leiden, The Netherlands; 5 Present Address: Department of Biochemistry and Molecular Biology, University of Texas Health Science Center at Houston, Houston, TX USA

Correction to: *Scientific Reports* 10.1038/s41598-019-52208-3, published online 29 October 2019

This Article contains errors in Figure 3, where the y-axis in Figure 3B has been incorrectly labelled. The correct Figure 3 appears below as Figure 1. As a result, the Figure legend,

**Figure 1:**
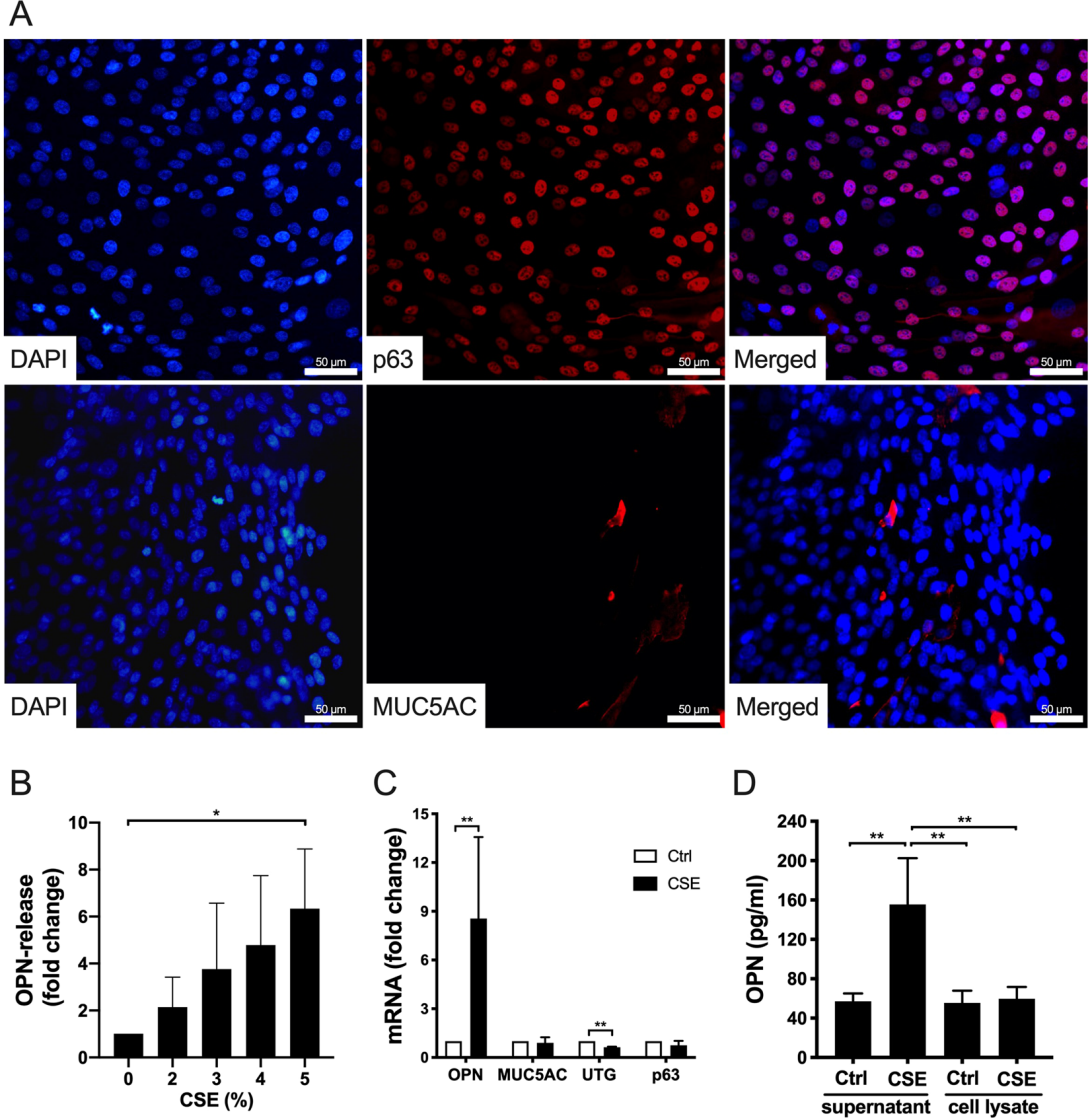
.

“Phenotypic characterization of submerged human bronchial epithelial cells (HBECs) and OPN production in response to cigarette smoke extract (CSE). **(A)** HBECs were grown to near confluence and stained to detect markers of basal cells (p63) and goblet cells (MUC5AC). 4′,6-Diamidino-2-phenylindole (DAPI) was used to stain the DNA of nuclei. Micrographs from one representative experiment out of three. Scale bars = 50 µm. **(B)** HBECs were incubated with CSE (0–5%) for 24 h. OPN levels in cell culture media were determined via enzyme-linked immunosorbent assay (ELISA). Results represent means and standard deviations (SD) of three independent experiments. Statistical analyses were performed using one-way ANOVA with Dunnett’s post-hoc test. **(C)** Submerged HBECs were cultured in the absence and presence of CSE (5%) for 24 h. The mRNA expression of OPN, MUC5AC, UTG, and p63 (fold-change compared to control cells cultured in medium alone) is depicted here. The results represent means and SD of three to five independent experiments. Statistical analyses were performed using a Mann-Whitney U test. **(D)** Intracellular OPN content and content in the media of cells cultured in the absence and presence of CSE (5%) for 24 h. The results represent means and SD of three independent experiments. Statistical analyses were performed using one-way ANOVA with Tukey’s post-hoc test. *P < 0.05, **P < 0.01.”

should read:

“Phenotypic characterization of submerged human bronchial epithelial cells (HBECs) and OPN production in response to cigarette smoke extract (CSE). **(A)** HBECs were grown to near confluence and stained to detect markers of basal cells (p63) and goblet cells (MUC5AC). 4′,6-Diamidino-2-phenylindole (DAPI) was used to stain the DNA of nuclei. Micrographs from one representative experiment out of three. Scale bars = 50 µm. **(B)** HBECs were incubated with CSE (0–5%) for 24 h. OPN levels in cell culture media were determined using ELISA. OPN-release from unstimulated cells (0% CSE) were set to 1, and the other conditions were calculated as fold change. Results represent means and standard deviations (SD) of three independent experiments. Statistical analyses were performed using one-way ANOVA with Dunnett’s post-hoc test. **(C)** Submerged HBECs were cultured in the absence and presence of CSE (5%) for 24 h. The mRNA expression of OPN, MUC5AC, UTG, and p63 (fold-change compared to control cells cultured in medium alone) is depicted here. The results represent means and SD of three to five independent experiments. Statistical analyses were performed using a Mann-Whitney U test. **(D)** Intracellular OPN content and content in the media of cells cultured in the absence and presence of CSE (5%) for 24 h. The results represent means and SD of three independent experiments. Statistical analyses were performed using one-way ANOVA with Tukey’s post-hoc test. *P < 0.05, **P < 0.01.”

