# Supplementary material for: Osteopontin Expression in Small Airway Epithelium in Copd is Dependent on Differentiation and Confined to Subsets of Cells
Source: Sci Rep. 2019 Oct 29;9:15566. doi: 10.1038/s41598-019-52208-3 (PMC6820743; doi:10.1038/s41598-019-52208-3)
Supplement: Supplementary file 1 — Supplementary information [file 41598_2019_52208_MOESM1_ESM.pdf]

SUPPLEMENTARY INFORMATION

Title

Osteopontin expression in small airway epithelium in COPD is dependent on differentiation and confined to subsets of cells

Authors

Mohamad N. Ali, Michiko Mori, Tinne C.J. Mertens, Premkumar Siddhuraj, Jonas S. Erjefält, Patrik Önnerfjord, Pieter S. Hiemstra, Arne Egesten

Figure S1. Phenotypic characterization of OPN-expressing cells in small airways of a never smoking controls

Immunofluorescence was used to detect possible co-localization of OPN and mucin 5AC (MUC5AC) in goblet cells **(A)**, uteroglobin (UTG) in club cells **(B),** p63 in basal cells **(C)**, and forkhead box protein J1 (FOXJ1) in ciliated cells **(D)**. In the overlay, a high degree of co-localization was observed for OPN and MUC5AC in goblet cells and UTG in club cells. One representative donor out of three. Scale bars = 100 µm.

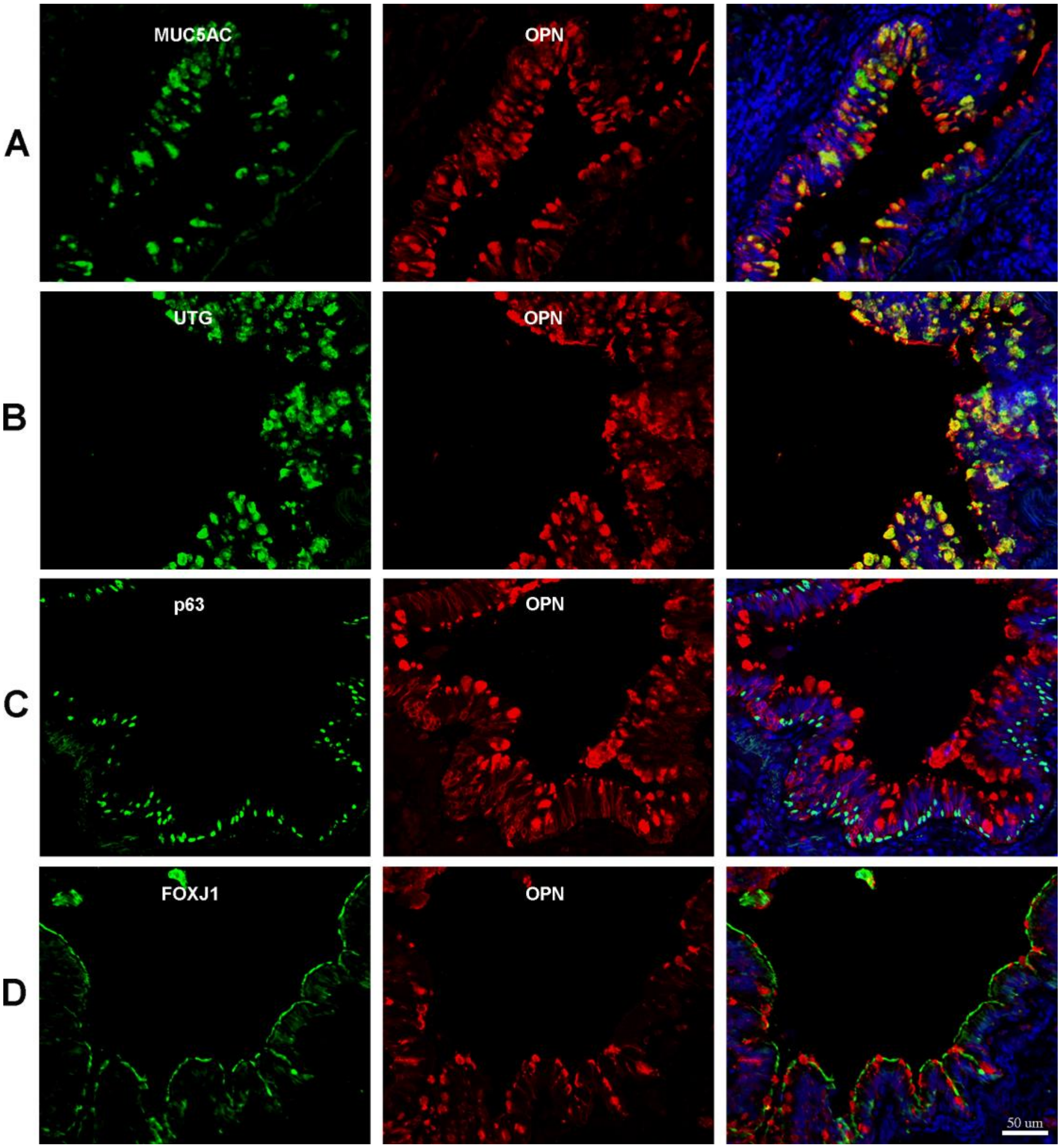

Table S1. Subject characteristics

Characteristic	Never-smokers	Smokers w/o COPD	GOLD I COPD	GOLD II-III* COPD	GOLD IV COPD
Subjects, n	8	6	6	12	9
Gender, male/female	2/6	2/4	4/2	9/3	4/5
Age, years	66 (33-76)#	57 (47-68)	68 (56-75)	66 (53-77)	62 (53-66)
Smoking history, pack-years	0	36 (20-80)	39 (25-66)	46 (30-65)	40 (25-60)
Smoking status, ex-smokers/current	NA	2/4	3/3	7/5	10/0
FEV_1	2.5 (1.7-5.1)	2.8 (1.9-3.5)	2.9 (1.6-3.2)	1.8 (1.2-2.9)	0.6 (0.4-1.0) □ □
FEV_1, % of predicted	109 (82-141)	95 (82-120)	86 (80-95)	67 (43-78)	24 (15-27) □ □ □
FEV_1/(F)VC, %	82 (66-121)	76 (71-88)	67 (65-70)	61 (41-68)	32 (20-39)
Inhaled β_2-agonists					
Short-acting (yes/no/unknown)	0/8/0	0/6/0	1/5/0	2/10/0	4/4/1‡
Long-acting (yes/no/unknown)	0/8/0	0/6/0	0/6/0	0/12/0	3/5/1‡
Inhaled anticholinergics					
Short-acting (yes/no/unknown)	0/8/0	0/6/0	1/5/0	2/10/0	2/6/1‡
Long-acting (yes/no/unknown)	0/8/0	0/6/0	0/6/0	0/12/0	5/3/1‡
Inhaled short-acting β_2-agonist plus anticholinergics (yes/no/unknown)	0/8/0	0/6/0	0/6/0	0/12/0	3/5/1‡
Corticosteroids					
Inhaled (yes/no/unknown)	0/8/0	0/6/0	0/6/0	1/11/0	2/6/1‡
Oral (yes/no/unknown)	0/8/0	0/6/0	0/6/0	0/12/0	2/6/1‡
Inhaled long-acting β_2-agonist plus corticosteroids (yes/no/unknown)	0/8/0	0/6/0	0/6/0	1/11/0	6/2/1‡
Mucolytics (yes/no/unknown)	0/8/0	0/6/0	2/4/0	0/12/0	5/3/1‡

Definition of abbreviations: COPD = chronic obstructive pulmonary disease; FEV_1 = forced expiratory volume in one second; (F)VC = (forced) vital capacity; GOLD = Global Initiative for Chronic Obstructive Lung Disease.

Values are median (range) or n.

* Two patients with GOLD Stage III COPD (median value of FEV_1% of predicted, 44.5%; range 43.2-45.9).

# The mean value of the study group is 63 years.

‡ One patient with unknown medical history.

Table S2. Primer sequences

Gene	Forward Primer	Reverse Primer
OPN	5'-GGACTCCATTGACTCGAACGACTCTG-3'	5'-AACCACACTATCACCTCGGCCATC-3'
FOXJ1	5'-GGAGGGGACGTAAATCCCTA-3'	5'-TTGGTCCCAGTAGTTCCAGC-3'
MUC5AC	5'-ATTTTTTCCCCACTCCTGATG-3'	5'-AAGACAACCCACTCCCAACC-3'
p63	5'-CCACCTGGACGTATTCCACTG-3'	5'-TCGAATCAAATGACTAGGAGGGG-3'
UTG	5'-CCCTGGTCACACTGGCTCTCTGC-'3	5'-CCCCTGCCTCCCTCATGTCTTG-'3
RPL13A	5'-AAGGTGGTGGTCGTACGCTGTG -3'	5'-CGGGAAGGGTTGGTGTTCATCC-3'

OPN, osteopontin; FOXJ1, forkhead box protein J1; MUC5AC, mucin 5AC; p63, tumor protein p63; UTG, uteroglobin; RPL13A, ribosomal protein L13A.
